# Writing as formation: supervising AI use in early-career medical authorship

**DOI:** 10.3389/frai.2026.1874377

**Published:** 2026-08-21

**Authors:** Tiago Jacinto

**Affiliations:** RISE-Health, Faculty of Medicine, University of Porto, Porto, Portugal

**Keywords:** academic writing, artificial intelligence, doctoral training, medical education, research integrity, supervision

## Abstract

International editorial bodies require authors to disclose the use of large language models in manuscript preparation and to retain accountability for the final text. This guidance is appropriate for formed researchers but does not address what trainees should or should not do during the formative phase of a doctoral thesis, a residency case report, or a first-authored paper. I propose a supervisor-led framework for AI use in medical doctoral and early-career scientific writing, grounded in an argument about professional formation rather than compliance alone; the scope is the writing and interpretation of results, not AI use across all of doctoral research. Writing is one of the settings in which scientific reasoning is exercised and tested, and disclosure is insufficient as a primary control during training because it addresses the reader rather than the writer. When a trainee offloads the first draft of an argument to a model, the formative work that drafting was meant to do does not happen, and the trainee risks becoming a copy-editor of plausible text rather than the author of its argument, a gap no disclosure recovers. The framework rests on three operational moves: a career-stage axis, a sequencing rule, and a revision trace. The career-stage axis makes the same AI operation potentially appropriate for a formed researcher and inappropriate for a first-year trainee. The sequencing rule, applied during training, permits generative AI use only after an unassisted first draft, so that the cognitive work the writing was meant to occasion is performed before the tool touches the text. The revision trace pairs a short disclosure paragraph with an internal log that makes the trainee's tool use inspectable to the supervisor. These moves are organized by a four-tier task typology, related in shape to AI-use rubrics developed in general higher education, delivered as a one-page policy that supervisors or institutions can adapt. The framework is prescriptive in places; the restraint it recommends is formative, and supervisors rather than policy are its primary enforcement mechanism.

## 1 Introduction

The way medical doctoral trainees write has changed. Large language models have become a routine part of the manuscript workflow, used for grammar, reformulation, translation, summary, and in many cases content generation. The International Committee of Medical Journal Editors (ICMJE) and the Committee on Publication Ethics (COPE) have issued clear positions in response: language models cannot be listed as authors, their use must be disclosed, and human authors remain accountable for the final text (International Committee of Medical Journal Editors, 2026; COPE Council, 2024). Most major medical journals have aligned their instructions to authors with these principles.

How well disclosure works is itself contested. Comparative reviews of editorial policies find that journals diverge on what counts as reportable AI use and on where it must be declared, and that the detectors editors might rely on misclassify human text often enough to be unsafe as evidence (Yoo, 2025; Chigwada and Ngulube, 2026). Audits of the published record, though they rely on imperfect detectors, find a rising share of papers that appear to contain undisclosed AI-generated text, and several authors now argue that transparency, not detection, is the only workable control (Schneider et al., 2025; Vaishya et al., 2025). That debate is the backdrop for this paper. If disclosure is hard to verify even at the manuscript level, it is weaker still as the main control during training, where a supervisor needs to know which cognitive work the trainee did alone, not only that a tool was used.

This guidance is appropriate for formed researchers submitting work to peer-reviewed journals. It is not sufficient for the formative phase of medical training, where writing is one of the mechanisms through which scientific reasoning is formed. A doctoral student who discloses AI-generated discussion content is compliant with ICMJE. Whether they have learned to construct an argument from their own data is a different question, and one that current policy does not address. The argument that follows concerns writing and the interpretation of results, the parts of doctoral work in which the trainee composes and defends claims; AI use in study conceptualization, design, literature search, and data generation raises related questions I do not take up here.

In this Conceptual Analysis, I argue that supervisors of medical doctoral and early-career writers need a framework that operates below the manuscript level. The framework rests on an argument and three operational moves. The argument is that writing is part of how medical researchers are formed, and that offloading the cognitive work of writing during training has costs that disclosure cannot offset. The operational moves are a career-stage axis, which treats task-level risk for a trainee differently from task-level risk for a formed researcher; a sequencing rule, which during training permits generative AI use only after an unassisted first draft of the same text; and a revision trace, which makes the trainee's tool use inspectable to the supervisor. A four-tier task typology, related in shape to AI-use rubrics developed in general higher education (Perkins et al., 2024), organizes these moves into a one-page policy that institutions can adopt. The tier count is scaffolding; the axes and the trace are where the framework differs from what already exists.

The framework is prescriptive in places. Trainees are not asked to abandon AI tools; supervisors are asked to structure the curriculum so that specific cognitive acts are performed without AI during formation, and that AI use is introduced deliberately, with disclosure, once those acts have been practiced.

## 2 The guidance and what it leaves undone

The ICMJE recommendations are explicit on authorship. Large language models cannot meet the authorship criteria because they cannot be held accountable for the work (International Committee of Medical Journal Editors, 2026). Authors must disclose AI use, review AI-generated outputs for accuracy, and take responsibility for the entire text. COPE has issued a compatible position, adding that editors should require disclosure and consider AI use when evaluating integrity (COPE Council, 2024). Editorials in JAMA and The Lancet have endorsed broadly similar positions, with variation in the detail of what must be disclosed and where (Flanagin et al., 2023; Bagenal, 2024). The launch of NEJM AI as a dedicated journal, framed by its inaugural editor around AI's growing role in medical knowledge production, signals the depth of institutional engagement even while a unified cross-journal policy remains unsettled (Kohane, 2024). An empirical bibliometric analysis of the top 100 publishers and the top 100 medical journals documented substantial heterogeneity in whether, and how, these principles appeared in instructions to authors (Ganjavi et al., 2024).

This guidance does useful work. It clarifies that responsibility for what the manuscript says rests with a human. It closes the door on AI authorship. It signals to readers that an AI tool has been involved in the text they are reading. For a reader evaluating the reliability of a published paper, disclosure is the right primary control.

Disclosure is the wrong primary control for training. The reasons are practical and pedagogical.

Practically, disclosure is a manuscript-level declaration. It tells the reader what was done but gives the supervisor no detail about which tasks the trainee performed and which were outsourced. A disclosure that says “Claude was used for language editing and discussion drafting” is compliant with ICMJE and uninformative for the supervisor trying to assess what the trainee has learned. The supervisor needs task-level information that current guidance does not require.

Pedagogically, the guidance presumes that the author is a formed researcher whose judgment is mature enough to evaluate AI output critically. For a formed researcher, this is reasonable. For a first-year doctoral student, it is not. Evaluating an AI-generated discussion section presupposes the ability to construct an alternative discussion section. That ability is what the training is meant to develop. A trainee who leans on the model to produce the first draft of a discussion has not practiced the act the evaluation depends on, and is at risk of mistaking plausibility for validity.

The same gap appears in every phase of formation that involves first-authored writing. Master's theses, residency case reports, and early-career publications are all formative. In each of them, writing does more than communicate findings; it is where the writer tests whether the argument holds. Guidance pitched at the manuscript level cannot distinguish between a finished researcher using AI to accelerate familiar tasks and a trainee using it to skip unfamiliar ones.

Training therefore needs a layer below current guidance, not a replacement for it. Supervisors need a framework that shapes what trainees do and do not do with AI during the formative phase, built on an argument about what writing is for. The rest of this paper sets out that argument and then that framework.

## 3 Writing as thinking: what we risk losing

Sterk and Rabe (2008) argued that writing a paper is itself a form of scientific thinking, and that the discipline of putting an argument into words forces the author to examine whether the argument actually holds. I take this claim seriously. Its consequences for how we train medical researchers have not been worked through in the current guidance on AI use.

In medicine, the connection between writing and reasoning is close but specific. Clinical reasoning and scientific reasoning share a structure: both construct an argument from incomplete evidence, calibrate certainty against what the evidence supports, and report honestly what is not known. The claim here is bounded. Bedside clinical judgment is formed mainly through direct contact with patients and the review of clinical evidence, not through retrospective writing, and a clinician who writes awkwardly may still reason expertly. What writing forms is narrower: the scientific, argumentative reasoning by which a researcher builds and defends a claim from data. A doctoral trainee drafting a discussion section is practicing that reasoning, not merely recording a conclusion already reached. A resident composing a case report is constructing the kind of evidence-to-claim argument they will also construct when justifying a decision to a colleague. Writing is one of the settings in which this reasoning is exercised and tested, rather than a separate skill applied to it afterwards.

One objection deserves a direct answer: on this objection, writing only presents reasoning that has already happened, so a poor writer may be an excellent reasoner and the influence of writing on reasoning is overstated. For the finished manuscript this is correct, and for clinical judgment it is largely correct throughout. The claim defended here is developmental rather than about the finished text. During formation, drafting is not the transcription of a settled argument; it is the occasion on which the argument is first assembled, and the places where it will not assemble are where the trainee discovers that the reasoning is still incomplete. That discovery is the formative event, and a trainee who never drafts the argument unaided seldom reaches it. The objection holds for the expert and for the page; it does not hold for the novice at the moment of learning.

This is why the current framing around AI in medical writing is inadequate for training. The ICMJE, COPE, and most editorial positions treat AI use as a manuscript-level question: what the reader should be told, what the author should remain accountable for (International Committee of Medical Journal Editors, 2026). That framing is correct as far as it goes, but it answers the wrong question for the formative phase. For a trainee, the question is whether the trainee has done the work the manuscript was supposed to occasion. Honesty about the manuscript's inputs is necessary but a different question.

Consider a doctoral student who uses a language model to draft the discussion of their first paper. The model produces a plausible text: the findings are placed in context, limitations are acknowledged, future directions are proposed. The student reads it, agrees with it, revises a few sentences, and submits. The manuscript is disclosed appropriately. No rule has been broken. But the student has not learned to construct the argument they now claim to hold. When a reviewer asks why the findings should be trusted, the student has no internal model of the reasoning, only the output of one.

This is not a hypothetical concern. Work on cognitive offloading has described a general pattern in which tools that reduce task demand also reduce the depth of engagement with the underlying problem (Risko and Gilbert, 2016). A 2025 survey study of adult users reported a negative association between frequent AI-tool use and measured critical-thinking scores, with engagement in cognitive offloading as a partial mediator (Gerlich, 2025). An electroencephalographic study of participants writing short essays with and without a large language model reported reduced fronto-parietal connectivity and poorer later recall of the writers' own text in the LLM-assisted condition, a pattern the investigators described as “cognitive debt” (Kosmyna et al., 2025). The literature is early. Effect sizes are uncertain, generalization to doctoral medical writing is not yet shown, and the long-term trajectory is unknown. I do not think supervisors should wait for definitive evidence before acting. The argument is asymmetric in risk: if I am wrong, a cohort of trainees has written their first few papers with more effort than strictly necessary; if I am right, a cohort of trainees has been trained as editors of AI output rather than as authors of medical argument.

The pedagogical tradition this draws on is not new. Writing-to-learn research has documented for decades that the act of drafting, revising, and struggling with a text produces understanding that passive reading does not (Graham et al., 2020; Bangert-Drowns et al., 2004). The cognitive-psychology literature on desirable difficulties makes a parallel point: conditions that reduce effort during learning often reduce learning itself, while conditions that increase effort during learning tend to improve long-term retention and transfer (Bjork and Bjork, 2011). Medical education has absorbed these findings in other contexts, including case-based reasoning and reflective practice, but has not yet applied them to the question of AI-assisted writing.

The strongest objection to this line of argument is that AI-assisted drafting might accelerate formation rather than hollow it. On this view, a trainee who reads a well-structured AI-generated discussion is exposed to the shape of a good argument earlier than they would be through unassisted struggle, and a non-Anglophone trainee who offloads English syntax to the model redirects cognitive resources to the reasoning that actually matters. Both points have force. I do not accept the inference from the first, however: what the trainee reads is not what the trainee can produce, and the gap between recognition and generation is exactly where formation happens. The second point, the language-barrier concern, is a real one, and it is why the framework below places translation of a complete draft in Tier 2 rather than Tier 3. A trainee who drafts in Portuguese and translates with assistance is performing the reasoning; a trainee who asks the model to generate English prose from a bulleted sketch is not. The counterargument narrows the framework but does not defeat it.

I use language models in my own work, and I teach my students to use them. The argument here is narrower: disclosure is the wrong primary control for the doctoral and early-career phase, because it addresses the reader rather than the writer. What the writer becomes is the object of training, and the training is shaped by what the writer is asked to do alone. If the struggle of drafting a discussion section is where a clinician-scientist learns to hold an argument honestly, then outsourcing that struggle is a formative gap that no disclosure can recover. This motivates the framework proposed in the next section, which takes as given that trainees will use AI and asks only what they should do alone before they do.

## 4 A task-level typology of AI use in medical scientific writing

This section develops the paper's central conceptual contribution: a task-level typology of AI use in medical scientific writing, layered with the two axes and the revision trace introduced above. Rubrics that grade AI use by cognitive level are not new. The AI Assessment Scale developed in general higher education by Perkins et al. (2024) assigns tasks to levels ranging from “no AI” to “full AI,” and related scales have been proposed across professional and graduate education more broadly. The typology below is closely related in shape to these scales, with four levels rather than five and with medical-doctoral writing as the application. What is specific to this framework is not the tier count but the two axes layered across the typology, career stage and sequencing. Career stage treats the same task differently for trainees and for formed researchers; sequencing requires, during training, an unassisted first draft before any generative use on the same text. A revision trace makes those rules evaluable. The tier taxonomy is scaffolding; the axes and the trace carry the argument.

With that positioning in place, consider the tiers themselves. They are defined by the cognitive nature of the task, not by the technology. What matters is what the writer is being asked to do, and how closely the task is tied to the reasoning the training is meant to produce. Table 1 summarizes the typology as a one-page policy that supervisors or institutions can adopt or adapt.

**Table 1:** A one-page policy for AI use in medical doctoral and early-career scientific writing.

Tier	Representative tasks	During training (doctoral, master's, residency, early career)	Post-qualification
Tier 1: mechanical and stylistic edits on text the author has drafted	Grammar and spelling; reference and BibTeX formatting; table-caption reformatting; synonym suggestion; subject-verb checks in non-native English	Use freely. Disclose if substantial.	Use freely. Disclose if substantial.
Tier 2: restructuring of text the author has already written	Translating the author's complete draft into another language; compressing the author's abstract to a word limit; reordering paragraphs in a section already drafted	Permitted only after a complete first draft by the trainee. Disclose.	Use freely. Disclose.
Tier 3: generation of substantive argumentative content	First draft of an introduction, discussion, or literature review; interpretation of clinical findings from results; synthesis of a study rationale	Do not use AI for the first draft. Draft unassisted, then use AI to revise, find weaknesses, or test counterarguments. Disclose.	Use with disclosure and verification.
Tier 4: integrity violations at any career stage	Fabricated or unread references; quantitative or clinical claims not independently verified against primary sources; sections ghost-authored by AI and presented as the author's own	Prohibited.	Prohibited.

Accompanying requirements during training: the trainee maintains a revision trace recording, for each AI interaction of substance, the date, tier, prompt summary, output summary, and what was retained, what was changed, and why. The supervisor reviews the trace alongside the manuscript. The trainee includes a disclosure paragraph modeled on the template in Section 5.

Tier 1 covers mechanical and stylistic tasks with minimal cognitive content. Grammar and spelling correction on a paragraph the trainee has drafted. Cleaning up BibTeX entries. Reformatting a table caption to match a journal style. Suggesting synonyms. Checking subject-verb agreement in a non-native English text. These tasks carry no meaningful risk to formation. The trainee has already done the cognitive work, and the model is doing editing work comparable to what a language editor would do. Tier 1 use is appropriate during and after training, with standard disclosure when substantial.

Tier 2 covers tasks that involve some restructuring of text the trainee has already generated. Translating the trainee's Portuguese draft into English. Compressing the trainee's 400-word abstract to 250 words. Reorganizing the order of paragraphs in a methods section that the trainee has already written. These tasks require the model to make small editorial judgments, but the underlying argument and the evidence are the trainee's own. Tier 2 use is appropriate in both training and post-qualification, with disclosure. For trainees, I suggest one additional condition: Tier 2 operations should follow a complete first draft by the trainee, not replace it. A trainee who drafts in Portuguese and translates to English is performing Tier 2. A trainee who asks the model to generate an English abstract from a Portuguese bulleted outline is performing Tier 3.

Tier 3 covers tasks that generate substantive argumentative content. Drafting a discussion section from a results summary. Producing an introduction from a few keywords. Writing a literature review from a set of references. Generating interpretations of clinical findings. Synthesizing a rationale for a study design. These are the tasks where the cognitive work is the writing, and where formation is most at risk. For formed researchers, Tier 3 use is defensible with careful disclosure and verification. For trainees, I suggest that Tier 3 use should be restricted to the revision phase: the trainee produces the first draft unassisted, then may use the model to suggest reformulations of their own text, to identify weaknesses in their argument, or to propose counterarguments that the trainee evaluates. The model never writes the first version of a Tier 3 section during training.

Tier 4 covers uses that should not occur at any career stage. Fabricated references, or references the author has not read. Generated content presented as the author's own without disclosure. Clinical claims not independently verified against primary sources. Statistical claims produced by the model without corresponding analysis. These are failures of integrity rather than gradients of risk, and existing research-conduct guidance already addresses them. The tier is included here for completeness and because trainees encountering AI tools without supervision may not recognize that certain uses cross the integrity line.

Two features of the framework deserve emphasis, and together they are where the specific contribution sits.

One concerns evaluation: the tier of a task depends on what the trainee brings to it, not on the prompt. Asking a model to “improve this paragraph” can be Tier 1, Tier 2, or Tier 3 depending on how much of the paragraph exists, how much is changed, and whether the change affects argumentative content. Supervisors cannot evaluate AI use from the prompt alone; they need the revision trace described in the next section. Evaluation must be trace-based rather than prompt-based, which in turn requires the artifact described below.

The other concerns sequencing during training. The typology permits Tier 3 use during training, but only under a temporal constraint: the trainee drafts the discussion first, using their own reasoning and their own prose. Once a complete draft exists, the trainee may use AI tools to revise it, to identify weaknesses, or to explore alternative framings. Draft first, then tool use, then disclosed tool use as standard practice in later career. Removing the struggle from the first step removes the formation. Compared with level-based scales in general education, which tend to fix the permitted AI level for a given assessment, the sequencing rule adds a temporal constraint on the same writer within the same artifact. Together, the trace-based evaluation and the sequencing rule are what adapt a general-education scale to the medical-doctoral setting.

Supervisors considering adoption should expect that the typology will produce edge cases the framework does not settle cleanly. A trainee who writes a discussion unassisted, then asks a model to produce an alternative version and selects phrases from both, is performing neither a pure Tier 3 first draft nor a simple Tier 2 revision. I would classify this as Tier 2 with the sequencing rule honored, on the grounds that the trainee produced the first draft and performed the evaluative work of comparison. A supervisor reading the revision trace is the right person to decide. The typology is a starting point for judgment, not a substitute for it.

## 5 Disclosure and the revision trace

Disclosure at submission is necessary but, as I have argued, insufficient for training. I propose two artifacts that operationalize the framework during the formative phase: a disclosure paragraph adapted for trainees, and a revision trace maintained for supervisor review.

The disclosure paragraph follows the structure already converging across major journals, with additions appropriate to training. It should specify the model and version, the date range of use, the tasks performed, the verification step, and the person accountable. A template, for a doctoral manuscript:

During the preparation of this manuscript, I used [model name and vendor; version identifier; date range of use] for Tier 1 language editing of [sections edited] and for Tier 2 [describe restructuring performed]. The [sections drafted unassisted] were written without AI assistance. All AI-suggested edits were reviewed against the source material and either accepted, revised, or rejected. I am accountable for the final text.

This template communicates more than a compliance declaration. It tells a reader and a supervisor what the trainee did unassisted and what the trainee did with help. It makes the structure of the work inspectable.

The revision trace is internal to the supervisor-trainee relationship and does not appear in the published manuscript. It is a log maintained by the trainee during the writing phase. For each AI interaction of any substance, the trainee records the date, the task category (Tier 1 to 3), a summary of the prompt, a summary of the model's output, what was retained, what was changed, and why. The trace does not need to be verbose. A handful of lines per entry is sufficient.

The revision trace does two things. It gives supervisors the information they need to judge what the trainee has learned. A trace dominated by Tier 3 generation and minimal revision is a training signal: the trainee has been using the model to avoid the work that forms their judgment. A trace dominated by Tier 1 and Tier 2 operations, with Tier 3 interactions that show the trainee evaluating, rejecting, and rewriting model output, is a different signal. The trace makes this distinction visible.

The trace is also itself formative. The act of documenting an AI interaction forces the trainee to articulate what the model did and what they did with it. It is harder to mistake a plausible AI discussion for one's own reasoning when one has to describe, in writing, what was retained and what was rejected. The trace is a metacognitive prompt disguised as a compliance instrument.

Neither artifact requires new infrastructure. The disclosure paragraph goes in the manuscript, where existing guidance already expects a similar declaration. The revision trace can be a plain text file, a shared document, or a structured form adopted at the institutional level. Institutional AI-governance policies are under active discussion at several medical schools; templates of the kind described here can be adapted to local training structures as those policies take shape.

Supervisors who adopt this approach should expect resistance. The revision trace adds work to a phase of training that trainees often find difficult enough already. The argument for the trace is the same as the argument for the framework as a whole: the writing is the training, and the evidence of what the trainee did during writing is the evidence of what the trainee learned. A training program that does not generate this evidence is training something, though not reliably what it intends to train.

## 6 Recommendations for doctoral writing instruction in medicine

The framework yields a small set of concrete recommendations for supervisors and programs:

- *State the sequencing rule at the start of each project* and revisit it at every manuscript: draft without AI, revise with AI, disclose.
- *Read the trainee's unassisted first draft* before any AI tool has touched it.
- *Require a revision trace* for AI interactions of substance, and review it alongside the manuscript.
- *Train supervisors* to read traces and to distinguish Tier 2 from Tier 3 operations, through a short workshop on the typology.
- *Make the argument visible to trainees* as a formative expectation open to challenge, not a policing rule.
- *Revise the tier boundaries at a regular interval*, with trainee input, as models change.

The paragraphs that follow expand the recommendations that need it. Stating the sequencing rule at the start of a thesis project, and revisiting it at each manuscript, prevents the drift that occurs when trainees adopt AI tools informally and retrospectively describe their use in whatever terms the journal requires. The practical complement to the rule is a small change in supervision workflow: read the unassisted first draft before the trainee has had the model touch it. An assisted first draft is partly shaped by a model whose reasoning the supervisor cannot see. An unassisted draft, however rough, reveals the trainee's own argument and lets the supervisor respond to that argument rather than to its polished AI shadow.

The framework assumes that supervisors themselves can distinguish Tier 2 from Tier 3 operations, read a revision trace with judgment, and hold a considered position on their own AI use. Many cannot, yet. A trainee who submits a trace documenting five rounds of “model suggested counterargument, I revised, I kept two sentences, I rejected three” is transparent; the supervisor has to be able to read that trace and know whether it describes appropriate use or a Tier 3 operation retrospectively decomposed into Tier 2 fragments. Institutions that adopt a framework of this kind should pair it with a short supervisor workshop on the typology and on reading revision traces. A framework that supervisors have not been trained to apply will produce variable and often arbitrary practice.

The argument and the framework should be visible to trainees, not only applied to them. Trainees are more likely to accept restraint during formation when the reason is explained and the reason is respectful of their autonomy. Explaining that the sequencing rule exists to protect the formation of their scientific judgment, and that the supervisor's expectation of unassisted first drafts is a formative expectation rather than a policing one, matters for how the rule is received. The framework is prescriptive, but the prescription is accountable to an argument the trainee can examine and challenge.

## 7 Limitations and open questions

The argument rests on two claims that currently lack strong empirical support in the specific context of medical doctoral writing: that writing is among the primary mechanisms through which medical scientific judgment is formed, and that offloading the cognitive work of writing during training materially affects that formation. The writing-to-learn literature, the cognitive psychology of desirable difficulties, and early evidence on cognitive offloading in AI-assisted work all point in this direction, but the specific question for medical doctoral training has not been studied directly. Supervisors adopting the framework do so on the asymmetric-risk argument set out above rather than on a demonstrated effect.

The framework is a conceptual proposal and has not been evaluated. Its applicability across specialties and settings, its validity as a way of characterizing formative AI use, and the reliability with which different supervisors would classify the same interaction are all untested. I have used elements of it in my teaching but have not collected data on trainee outcomes, supervisor adoption, or long-term effects on published work. A modest empirical program would be valuable: prospective cohorts of trainees under different AI-use regimes, assessed on later first-authored publications and on supervisor evaluations of argumentative independence, together with inter-rater studies of how consistently supervisors apply the tiers.

The typology simplifies a domain that resists simplification. Subspecialties within medicine vary. The pace of model change will move boundaries. Some tasks I have classified as Tier 2 may become Tier 1 within 2 years, and institutions that adopt the framework should plan to revise it, with trainee input, at a regular interval. A framework that does not evolve will become either an obstacle or a dead letter, and neither outcome supports formation.

## 8 Conclusion

Medical doctoral training produces researchers, not manuscripts. Manuscripts are the evidence that the training has happened, and writing is one of the mechanisms through which it happens. Current guidance on AI use addresses the manuscript and the reader, which is appropriate for formed researchers. For trainees, the supervisor needs a layer below this: a task typology organized by career stage and sequencing, a disclosure practice that informs, and a revision trace that makes the trainee's cognitive work visible. The framework applies to the writing and interpretation tasks of formation, the discussion drafted, the case report composed, the results interpreted, and not to every use of AI across a doctoral project. Within that scope, the restraint it proposes is formative.
